# MicroRNA‐21 induces 5‐fluorouracil resistance in human pancreatic cancer cells by regulating *PTEN* and *PDCD4*

**DOI:** 10.1002/cam4.626

**Published:** 2016-02-10

**Authors:** Xueju Wei, Weibin Wang, Lanlan Wang, Yuanyuan Zhang, Xian Zhang, Mingtai Chen, Fang Wang, Jia Yu, Yanni Ma, Guotao Sun

**Affiliations:** ^1^ Institute of Molecular MedicineMedical SchoolHenan UniversityKaiFeng475000China; ^2^ Department of Biochemistry and Molecular BiologyInstitute of Basic Medical SciencesChinese Academy of Medical SciencesSchool of Basic Medicine Peking Union Medical CollegeBeijing100005China; ^3^ Department of General SurgeryPeking Union Medical College HospitalCAMS & PUMCBeijing100005China; ^4^ The First Hospital Affiliated To Henan UniversityHenan475000China

**Keywords:** 5‐Fluorouracil, miR‐21, pancreatic cancer, PDCD4, PTEN

## Abstract

Pancreatic cancer patients are often resistant to chemotherapy treatment, which results in poor prognosis. The objective of this study was to delineate the mechanism by which miR‐21 induces drug resistance to 5‐fluorouracil (5‐FU) in human pancreatic cancer cells (PATU8988 and PANC‐1). We report that PATU8988 cells resistant to 5‐FU express high levels of miR‐21 in comparison to sensitive primary PATU8988 cells. Suppression of miR‐21 expression in 5‐Fu‐resistant PATU8988 cells can alleviate its 5‐FU resistance. Meanwhile, lentiviral vector‐mediated overexpression of miR‐21 not only conferred resistance to 5‐FU but also promoted proliferation, migration, and invasion of PATU8988 and PANC‐1 cells. The proresistance effects of miR‐21 were attributed to the attenuated expression of tumor suppressor genes, including *PTEN* and *PDCD4*. Overexpression of *PTEN* and *PDCD4* antagonized miR‐21‐induced resistance to 5‐FU and migration activity. Our work demonstrates that miR‐21 can confer drug resistance to 5‐FU in pancreatic cancer cells by regulating the expression of tumor suppressor genes, as the target genes of miR‐21, *PTEN* and *PDCD4* can rescue 5‐FU sensitivity and the phenotypic characteristics disrupted by miR‐21.

## Introduction

Pancreatic cancer is a highly lethal malignant disease and has a poor prognosis, despite the development of medical and surgical management. Incidence and mortality are nearly identical for pancreatic cancer. Pancreatic ductal adenocarcinoma (PDAC) represents over 90% of all pancreatic malignancies and is usually diagnosed at later stages with limited therapeutic options. Only 20% of patients are candidates for surgical resection, which has a 5‐year survival rate in <5% of patients after postoperative treatment 1. Therefore, for advanced pancreatic cancer, chemotherapy has become the standard and most often used treatment 2. Gemcitabine and 5‐fluorouracil (5‐FU) are classic chemotherapeutic drugs that have been widely used for the treatment of pancreatic cancer. However, primary or acquired drug resistance often causes therapeutic failure. In contrast to the sizeable amount of research focusing on the mechanism of gemcitabine resistance, the mechanism of 5‐FU resistance is not well studied, even though its therapeutic potential was discovered first, and it is still commonly used to treat pancreatic cancer 3. Thus, there is an urgent need to understand the mechanism of 5‐FU resistance and to develop new strategies to overcome drug resistance in the chemotherapy of pancreatic cancer.

MicroRNAs (miRNAs) are small, noncoding molecules that can regulate gene expression by inhibiting translation initiation or accelerating mRNA degradation 4. Diverse biological processes require the function of miRNAs, and the dysfunction of miRNAs is associated with many diseases, including cancer 5. Oncogenic signaling, tumor growth and chemoresistance can all be regulated by mRNAs 6, 7. Moreover, accumulating evidence suggests that the deregulation of miRNAs contributes to resistance to anticancer drugs 3, 8.

We investigated the differentially expressed miRNAs between the 5‐Fu‐resistant cell line (PATU8988/5‐FU) and the primary PATU8988 cell line and found that miR‐21 levels were significantly increased in 5‐FU‐resistant cells. As an oncogene, miR‐21 has been reported to promote drug resistance in various cancers 9. For example, overexpression of miR‐21 in breast cancer can promote taxol, trastuzumab, and doxorubicin resistance 10, 11, 12. The abnormal expression of miR‐21 in gastric cancer can result in cisplatin resistance 13. In addition, miR‐21 plays an important role in gemcitabine or 5‐FU resistance in pancreatic cancer as well as in other cancers 14, 15, 16. Previous research concludes that miR‐21 promotes drug resistance to treatments of these cancers and thus may be a new target for the improvement of clinical treatment. However, the role of miR‐21 in 5‐FU resistance and its mechanism in pancreatic cancer are not well understood.

In this study, we examined the role and mechanism of miR‐21 in promoting the 5‐FU resistance of pancreatic cancer cells. We first detected that miR‐21 was upregulated in 5‐FU resistance cell line PATU8988/5‐FU and down‐regulation of miR‐21 in PATU8988/5‐FU cells can alleviate its 5‐FU resistance. Introduction of miR‐21 into PATU8988 and PANC‐1 cells significantly enhanced their resistance to 5‐FU and also promoted proliferation, migration and invasion of PATU8988 and PANC‐1 cells. Further investigation indicated that the essential role of miR‐21 in 5‐FU resistance was dependent on its two targets, PTEN and PDCD4. Overexpression of PTEN and PDCD4 can attenuate the effects of miR‐21 on 5‐FU resistance in pancreatic cancer cells. These findings suggest that the resistance to 5‐FU in pancreatic cancer cells is mediated, at least in part, via a faulty miR‐21/PTEN/PDCD4 cascade.

## Materials and Methods

### Cell culture

Human pancreatic cancer cell lines (PATU8988 and PANC‐1) were obtained from the Department of General Surgery, Peking Union Medical College (Beijing, China), and 293TN cells were obtained from the American Type Culture Collection. PANC‐1 and 293TN cells were propagated in Dulbecco's modified Eagle medium (Invitrogen, Carlsbad, CA, USA) supplemented with 10% fetal bovine serum (Invitrogen), streptomycin (100 mg/mL) and penicillin (100 U/mL), while PATU8988 cells were maintained in RPMI 1640 medium (Invitrogen) supplemented with 10% FBS (Invitrogen). All cells were maintained at 37°C in humidified air with 5% CO_2_.

### Construction and transduction of recombinant lentivirus

The pMIRNA1 plasmid and pPACKH1 lentivector packaging kit were purchased from SBI (System Biosciences, Menlo Park, CA, USA). A 500‐bp DNA fragment flanking pre‐miR‐21 was inserted downstream of the CMV promoter in pMIRNA1 to generate pMIRNA1‐miR‐21. Viral packaging was performed according to the manufacturer's instructions. Virus particles (lenti_miR‐21 and lenti_GFP) were harvested and concentrated using PEG‐it Virus Precipitation Solution (SBI). Virus titer was determined in 293TN cells using a global ultrarapid lentiviral titer kit (SBI). For gene transduction into PATU8988 and PANC‐1 cells, the recombinant virus particles were added to the culture medium of the cells at an MOI = 3–5.

### Construction and transfection of expression plasmids

The coding sequence of *PDCD4* and *PTEN* were obtained and inserted into the pcDNA3.1 (+) plasmid. A 500‐bp DNA fragment flanking pre‐miR‐21 was inserted into the pcDNA3.1 (+) plasmid. The recombinant expression plasmids were transfected into PATU8988 and PANC‐1 cells using lipid reagents (Qiagen, Beijing, China). The transfection efficiency was confirmed by Western blot.

### Quantitative RT‐PCR

Total RNA was extracted using Trizol reagent. For miRNAs, the stem‐loop reverse transcription was manufactured according a previous report 17. Quantitative RT‐PCR was performed using the Bio‐Rad CFX‐96 system (Bio‐Rad, San Francisco, CA, USA) with the SYBR Premix ExTaq kit (Takara, Dalia, China). The data were normalized using the endogenous U6 snRNA for miRNAs. The 2^−ΔΔCT^ method was used to determine the relative expression of RT‐PCR data.

### In vitro cytotoxicity tests

5‐Fluorouracil was purchased from Eli Lilly and Company (Indianapolis, Indiana State, USA). PATU8988 cells were plated in triplicate at 8 × 10^3^ cells per well in 96‐well plates. In the same manner, PANC‐1 cells were plated in triplicate at 1 × 10^4^ cells per well in 96‐well plates. Four hours later, 5‐FU (fourfold serial dilution, from 1 × 10^3^ to 9.54 × 10^−4^ *μ*g/mL) was added and incubated for 72 h.

### Cell proliferation assays

PATU8988 cells were seeded into 96‐well plates at 2.5 × 10^3^ cells per well, and PANC‐1 cells were seeded into 96‐well plates at 5 × 10^3^ cells per well. The cells were incubated with 10 *μ*L CCK‐8 (Dojindo, Tokyo, Japan) for 2 h. Proliferation rates were determined at 0, 24, 48, 72, 96 h after seeding. All experiments were performed at least three times.

### Cell wound‐healing assay

PATU8988 and PANC‐1 cells were grown to confluence in 24‐well plates. To decrease the influence of cell proliferation in response to wounding, cells were maintained in medium containing 2% serum. Linear scratch wounds (in triplicate) were created on the confluent cell monolayers using a sterile 200 *μ*L pipette tip; images were taken at 0, 12, 24, 36 h.

### Cell migration assay

After adjusting the PATU8988 cells to a concentration of 1× 10^6^/mL and PANC‐1 cells to 5× 10^5^/mL, 0.1 mL cell suspensions of each group were seeded into the upper compartment of the chambers, and medium containing 20% serum was added to the lower chamber as a chemoattractant. Chambers were incubated at 37°C. After 24 h, supernatant was discarded and cells in the upper chamber were gently removed using a cotton swab. The cells in the lower surface were fixed with paraformaldehyde and stained with 0.1% crystal violet (Sigma, San Francisco, CA, USA). A total of eighteen areas were randomly selected from each well, and the cells in three wells of each group were quantified.

### Cell invasion assay

PATU8988 and PANC‐1 cells were seeded onto a Matrigel coated membrane matrix (BD Bioscience, NY, USA) present in the insert of a 24‐well culture plate. Fetal bovine serum was added to the lower chamber as a chemoattractant. After 24 h, the noninvading cells were gently removed with a cottons swab. Invasive cells located on the lower surface of chamber were stained with the 0.1% crystal violet and counted.

### Western blotting

PATU8988 and PANC‐1 cells were harvested in ice‐cold PBS and lysed in radioimmunoprecipitation assay (PIRA) buffer supplemented with protease inhibitors. Protein concentration was determined using the BCA protein assay kit (Rockford, IL, USA) and equal amounts of proteins were analyzed using SDS–PAGE (10% acrylamide gel). The antibodies used for Western blot were a *GAPDH* rabbit mAb antibody (Santa Cruz Biotechnology San Francisco, CA, USA) and *PDCD4* and *PTEN* rabbit mAb antibodies (Cell Signal Technology, Danvers, MA).

### Statistics

For comparison between 2 groups, Student's *t*‐test (two‐tailed) was used, and *P* < 0.05 were considered significantly different, as indicated by an asterisk (*P < 0.05; **P < 0.01; ***P < 0.001).

## Results

### Identification of miR‐21 associated with 5‐FU resistance in PATU8988 cells

**Figure 1:**
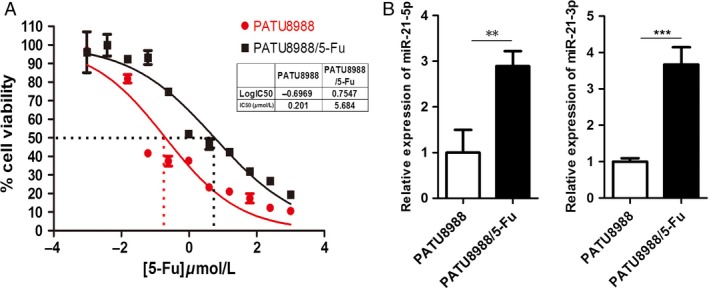
IC50s and expression of miR‐21 in PATU8988/5‐FU and PATU8988 cells. (A) Representative curves of growth inhibitory in 5‐FU‐resistant PATU8988/5‐FU and PATU8988 cells. IC50, half maximal inhibitory concentration. (B) The expression of miR‐21‐5p and miR‐21‐3p in 5‐FU‐resistant PATU8988/5‐FU cells and its parental PATU8988 cells. The error bars represent the standard deviation obtained from three independent experiments. ***P < 0.001 **P<0.01.

We established a 5‐FU‐treated PATU8988 cell line by increasing the 5‐FU concentration every 2 weeks for 6 months. IC50 was determined to be 0.201 *μ*mol/L for PATU8988 cells and 5.684 *μ*mol/L for PATU8988/5‐FU cells. Thus, the resistance of PATU8988/5‐FU cells to 5‐FU was estimated to be approximately ten times higher than that of the parental cells (Fig. 1A). To further study the role of miRNAs, we investigated the miRNA expression profile of PATU8988/5‐FU and PATU8988 cells using microarrays. We analyzed the upregulated expression and downregulated expression of miRNAs. As a result, the level of miR‐21 was strikingly upregulated in 5‐FU‐resistant cells compared to the parental cells. We further validated the expression of miR‐21 using quantitative RT‐PCR. The results indicated that the expression levels of miR‐21‐5p and miR‐21‐3p were upregulated in the PATU8988/5‐FU cells compared with expression in the PATU8988 cells (Fig. 1B). These results led us to speculate that the deregulation of miR‐21 may be associated with the 5‐FU‐resistance of pancreatic cancer cells.

### Increased intracellular miR‐21 level induces resistance to 5‐FU

**Figure 2:**
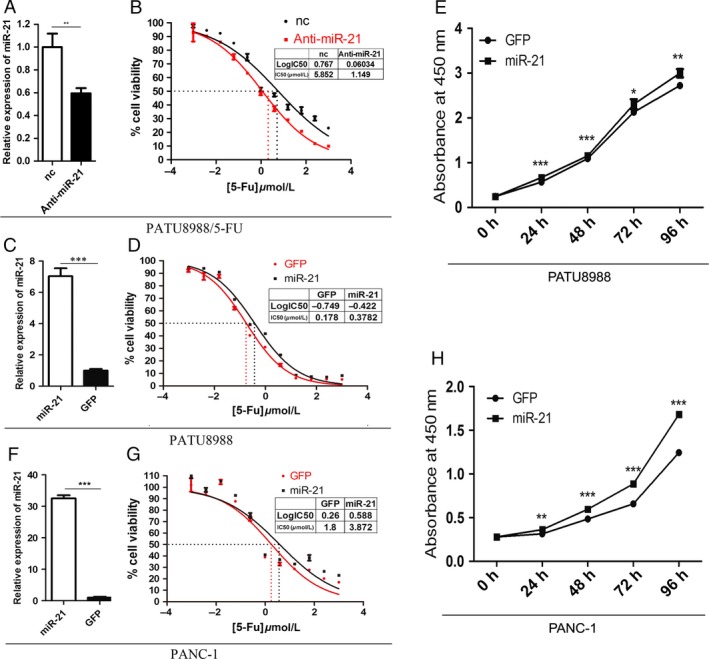
Cytotoxicity activity and proliferation rates of pancreatic cancer cells with miR‐21 overexpression. (A) The expression of miR‐21 in PATU8988/5‐FU cells transfected with negative control (nc) or miR‐21 inhibitor (anti‐miR‐21). (B) Representative curves of growth inhibitory in PATU8988/5‐FU cells transfected with negative control(nc) or miR‐21 inhibitor. (C) RT‐PCR showed the expression of miR‐21 in PATU8988 cells infected with lenti_miR‐21 and control group lenti_GFP. (D) Representative curves of growth‐inhibitory effects of 5‐FU in PATU8988 cells after treatments. (E) The expression of miR‐21 in PANC‐1 cells. (F) Representative curves of growth inhibitory effects of 5‐FU in PANC‐1 cells. (G) The cell growth curve of PATU8988 cells. (H) The cell growth curve of PANC‐1 cells. *P < 0.05, ** *P* < 0.01 and ***P < 0.001.

To understand the relationship between miR‐21 and 5‐FU resistance in pancreatic cancer cells, we transfected miR‐21 inhibitors in PATU8988/5‐FU to specifically inhibit the expression of miR‐21. The suppressed expression level of miR‐21 was determined by quantitative RT‐PCR (Fig. 2A). As a result, anti‐miR‐21 increased sensitivity to 5‐FU in PATU8988/5‐FU cells and IC50 was determined to be 1.149 *μ*mol/L for anti‐miR‐21 group and 5.852 *μ*mol/L for nc group (Fig. 2B). To further examine the contribution of miR‐21 to the resistance to 5‐FU in pancreatic cells, we transduced lenti_miR‐21 in its parental cell line PATU8988 to overexpression miR‐21 and treated with 5‐FU. MicroRNA‐21 was overexpressed successfully in PATU8988 cells, as determined by quantitative RT‐PCR (Fig. 2C). As a result, miR‐21 decreased sensitivity to 5‐FU in PATU8988 cells compared to lenti_GFP group (as a control). IC50 was determined to be 0.3782 *μ*mol/L for the miR‐21 overexpression group and 0.178 *μ*mol/L for the control in PATU8988 cells (Fig. 2D). Thus, the IC50 values of miR‐21 overexpressed cells were approximately 2 times higher than the values of the control cells. To confirm our conclusion, we detected another pancreatic cell line PANC‐1 in the similar way and we received analogous results. The expression of miR‐21 in PANC‐1 cells was determined by quantitative RT‐PCR (Fig. 2E) and cell cytotoxicity assay was tested by CCK8. IC50 was determined to be 3.872 *μ*mol/L for the miR‐21 overexpression group and 1.8 *μ*mol/L for the control in PANC‐1 cells (Fig. 2F). Furthermore, the proliferation rate of pancreatic cells was measured at 0, 24, 48, 72, 96 h and the results showed that miR‐21 promoted the growth of both pancreatic cell lines (Fig. 2G and H).

### MiR‐21 promotes migration and invasion of PATU8988 cells

**Figure 3:**
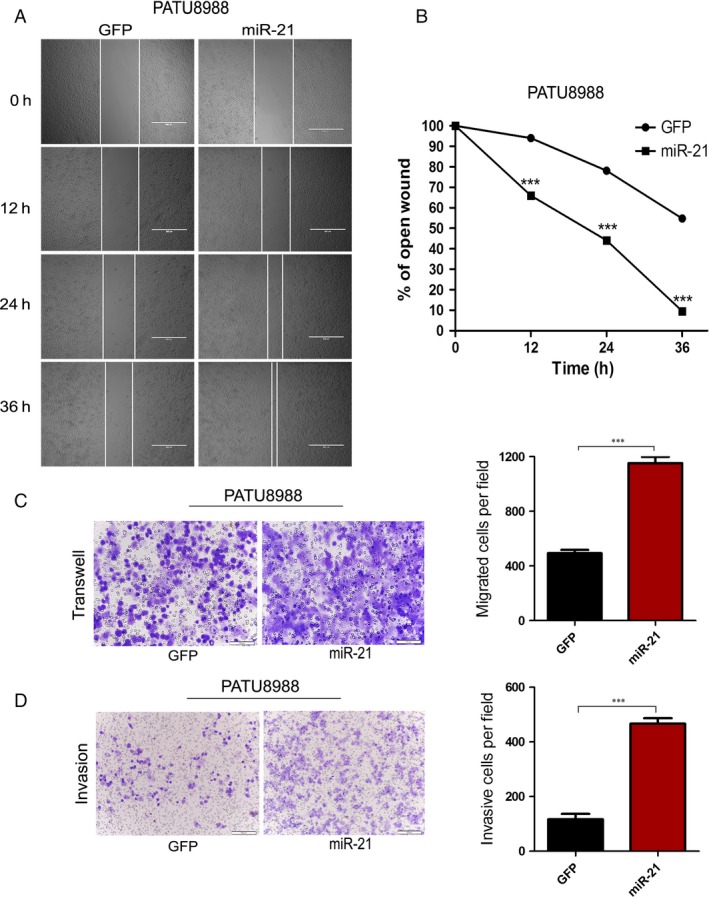
Overexpression of miR‐21 promotes PATU8988 cells migration and invasion. (A) The pictures of wound healing of PATU8988 cells infected with lenti_miR‐21 or lenti_GFP at times 0, 12, 24 and 36 h from the scratch. (B) The relative ratio of wound closure per field at each time point is shown. (C) Cells migrated to the bottom of membranes were stained and photographed. (D) Matrigel cell invasion assay. In (C and D), microscopic magnification (100×). The relative ratio of invasive cells per field is shown. ***P < 0.001.

To further determine the relationship between miR‐21 and other resistance‐associated phenotypes, such as cell migration and invasion, wound‐healing, transwell migration and invasion assays were performed. The results showed that overexpression of miR‐21 stimulated the migration of PATU8988 cells. The percentage of wound healing at 0, 12, 24 and 36 h time points after scratching was significantly increased in PATU8988 cells with miR‐21 overexpression (Fig. 3A and B). A similar phenomenon was observed in the migrated cells (Fig. 3C). In addition, we conducted a Matrigel cell invasion assay to assess the role of miR‐21 in regulating cell invasion. The results showed that overexpression of miR‐21 dramatically facilitated the invasion of PATU8988 cells (Fig. 3D). These results suggest that miR‐21 promotes cell migration and invasiveness in PATU8988 pancreatic cells.

### MiR‐21 promotes migration and invasion of PANC‐1 cells

**Figure 4:**
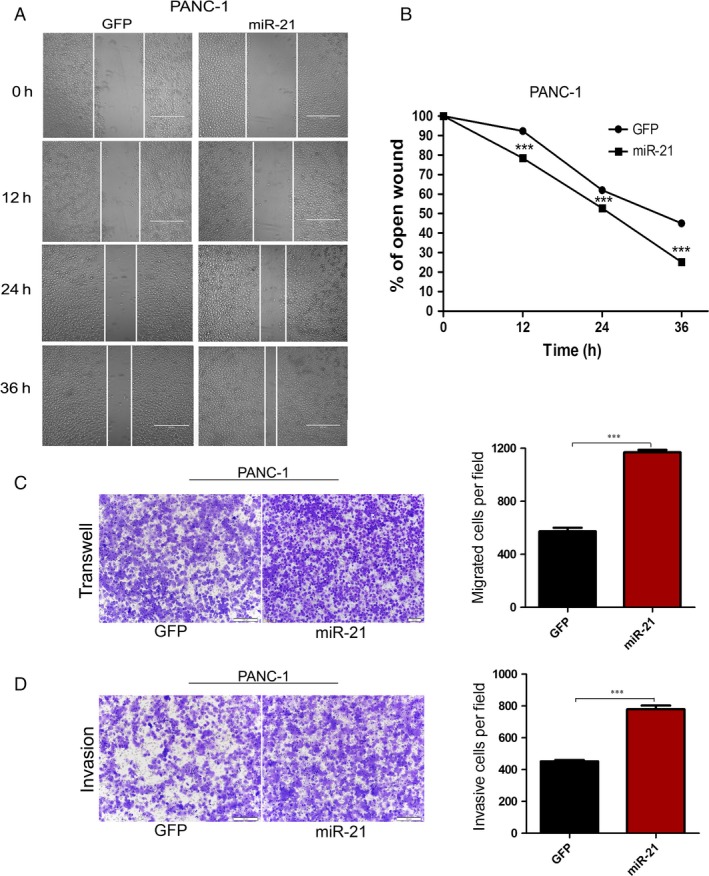
Overexpression of miR‐21 promotes PANC‐1 cells migration and invasion. (A) The pictures of wound healing of PANC‐1 cells infected with lenti_miR‐21 or lenti_GFP at times 0, 12, 24 and 36 h from the scratch. (B) The relative ratio of wound closure per field at each time point is shown. (C) Cells migrated to the bottom of membranes were stained and photographed. (D) Matrigel cell invasion assay. In (C and D), microscopic magnification (100×). The relative ratio of invasive cells per field is shown. ***P < 0.001.

We also analyzed the effect of miR‐21 on the migratory and invasive behavior of PANC‐1 cell, using wound‐healing assays and transwell assays. We found that miR‐21 significantly promoted migration during the closing of a wound created in a confluent monolayer (Fig. 4A and B). Similarly, transwell assays showed a stronger migratory capacity in PANC‐1 cell with miR‐21 overexpression than the control group (Fig. 4C). The invasion capacity was also significantly improved in PANC‐1 cells with miR‐21 overexpression (Fig. 4D). These results suggested that miR‐21 promoted cell migration and invasiveness in PANC‐1 pancreatic cells.

### MiR‐21 stimulates 5‐FU resistance through down‐regulation of *PTEN*

**Figure 5:**
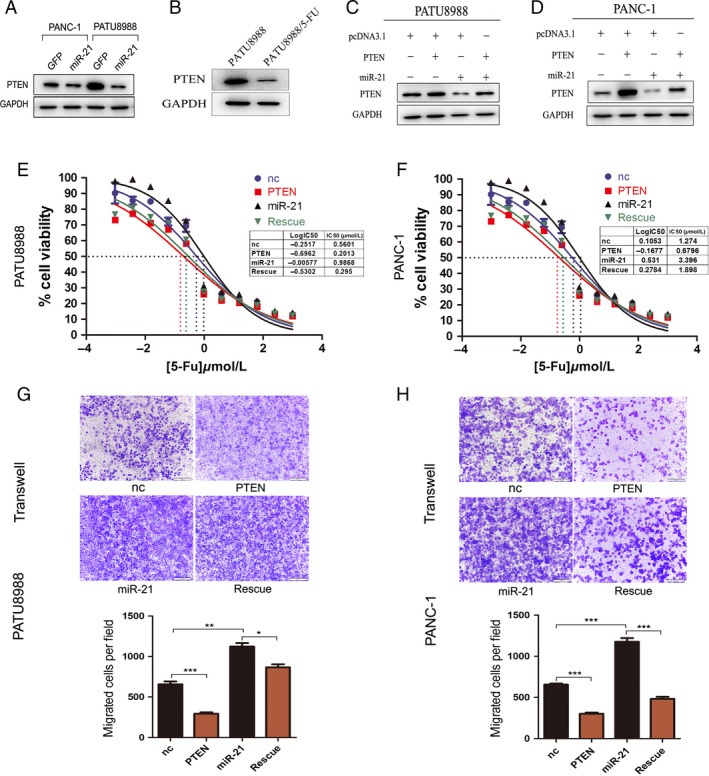
MiR‐21 promotes 5‐fluorouracil (5‐FU) resistant in pancreatic cancer by targeting *PTEN* directly. (A) Western blot showed *PTEN* protein levels in PANC‐1 and PATU8988 transfected with pcDNA3.1_miR‐21 or pcDNA3.1. (B) Western blot showed *PTEN* protein levels in PATU8988/5‐FU and PATU8988 cells. (C) Rescue assays by transfection with pcDNA3.1 (nc), pcDNA3.1_miR‐21 (miR‐21), pcDNA3.1_PTEN (*PTEN*) or pcDNA3.1_miR‐21 plus pcDNA3.1_PTEN (rescue) in PATU8988 and western blot. (D) Rescue assays and western blot in PANC‐1. (E) Representative curves of growth‐inhibitory effects of 72 h 5‐FU exposure in PATU8988 in rescue assay. (F) Representative curves of growth‐inhibitory effects of 72 h 5‐FU exposure in PANC‐1 in rescue assay. (G) Transwell assay after transfection in PATU8988 cells for 24 h. (H) Transwell assay after transfection in PANC‐1 cells for 24 h. In (G and F), microscopic magnification (100×). The relative ratio of invasive cells per field is shown. *P < 0.05, **P < 0.01 and ***P < 0.001.

*PTEN* has been reported as a target gene of miR‐21 in various cancers, including pancreatic cancer. To understand the mechanisms of miR‐21 in 5‐FU resistance, we first detected the expression level of *PTEN* in PATU8988 and PANC‐1 cell lines overexpressing miR‐21 and found that *PTEN* levels decreased significantly (Fig. 5A). We also detected the expression of *PTEN* in PATU8988 cell as well as its resistant cell line PATU8988/5‐FU. The results showed that PTEN was downregulated in PATU8988/5‐FU cells compared with its parental cells, which may be due to upregulation of miR‐21 in PATU8988/5‐FU cells (Fig. 5B). To further examine whether miR‐21 regulation of 5‐FU resistance is dependent on *PTEN* targeting, we employed a “rescue” experiment with miR‐21 and *PTEN* overexpression plasmids in PATU8988 and PANC‐1 cells. Transfection of *PTEN* alleviated the reduction in *PTEN* induced by miR‐21 treatment in the two pancreatic cancer cell lines (Fig. 5C and D). Consistent with the restored *PTEN* expression, miR‐21‐induced 5‐FU resistance was rescued in PATU8988 and PANC‐1 cells (Fig. 5E and F). Furthermore, overexpression of *PTEN* also attenuated the improved migratory ability induced by miR‐21 in both PATU8988 (Fig. 5G) and PANC‐1 (Fig. 5H) cells. These data confirm the regulatory role of miR‐21 on 5‐FU resistance through the targeting of *PTEN*.

### MiR‐21 stimulates 5‐FU resistance by regulating *PDCD4*

**Figure 6:**
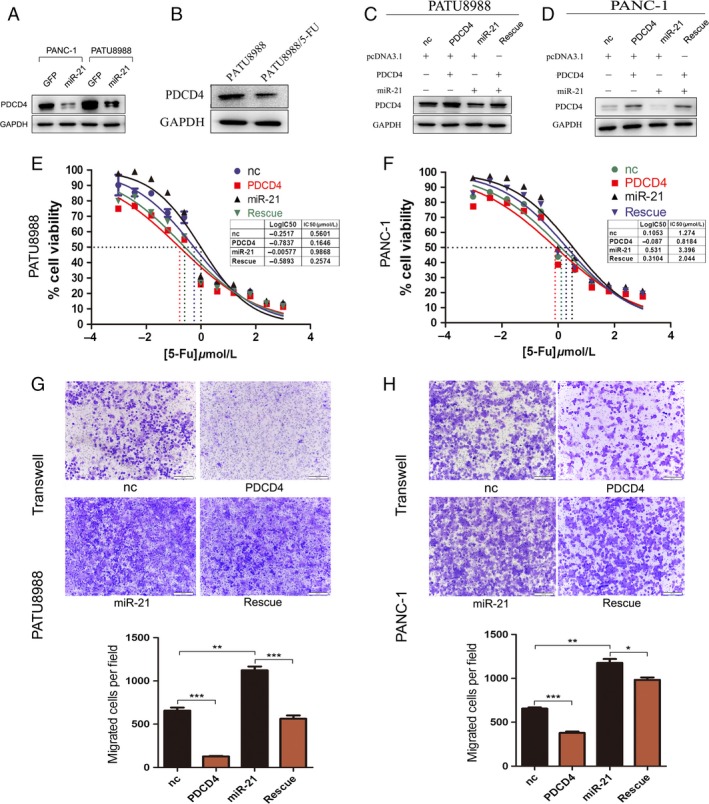
MiR‐21 promotes 5‐fluorouracil (5‐FU) resistant in pancreatic cancer by targeting *PDCD4* directly. (A) Western blot showed *PDCD4* protein levels in PATU8988 and PANC‐1 transfected with pcDNA3.1_miR‐21 or pcDNA3.1. (B) Western blot showed *PTEN* protein levels in PATU8988/5‐FU and PATU8988 cells. (C) Rescue assays by transfection with pcDNA3.1 (nc), pcDNA3.1_miR‐21 (miR‐21), pcDNA3.1_PDCD4 (*PDCD4*) or pcDNA3.1_miR‐21 plus pcDNA3.1_PDCD4 (rescue) in PATU8988 and western blot. (D) Rescue assays and western blot in PANC‐1. (E) Representative curves of growth‐inhibitory effects of 72 h 5‐FU exposure in PATU8988 in rescue assay. (F) Representative curves of growth‐inhibitory effects of 72 h 5‐FU exposure in PANC‐1 in rescue assay. (G) Transwell assay after transfection in PATU8988 cells for 24 h. (H) Transwell assay after transfection in PANC‐1 cells for 24 h. In (G and F), microscopic magnification (100×). The relative ratio of invasive cells per field is shown. *P < 0.05, **P < 0.01 and ***P < 0.001.

*PDCD4* is another target gene of miR‐21 in pancreatic cancer cells. In this study, transfection of PATU8988 and PANC‐1 cells with miR‐21 showed that *PDCD4* was significantly downregulated accompanied with miR‐21 overexpression (Fig. 6A). Furthermore, we detected the expression levels of PDCD4 in PATU8988/5‐FU cells and its parental cells, the data showed that PDCD4 was decreasedin 5‐FU resistance cell line which also may be due to up‐regulation of miR‐21 in PATU8988/5‐FU cells (Fig. 6B). To definitively determine whether miR‐21‐induced 5‐FU resistance was dependent on *PDCD4*, we performed rescue experiments with miR‐21 and *PDCD4* overexpression plasmids in the two pancreatic cancer cell lines. Overexpression of miR‐21 and *PDCD4* blocked both the reduction in *PDCD4* protein level and the enhancement of 5‐FU resistance that resulted from miR‐21 treatment in PATU8988 cells (Fig. 6C and E) and PANC‐1 cells (Fig. 6D and F). Similarly, we also performed migrated cells in PATU8988 and PANC‐1 cells. The results indicated that overexpression of *PDCD4* led to a decrease in cell migration induced by miR‐21 in both PATU8988 and PANC‐1 cells (Fig. 6G and H). All the data confirm that miR‐21 plays an important role in 5‐FU resistance in pancreatic cancer by targeting *PDCD4*.

Our studies demonstrate that the stimulating effect of miR‐21 on 5‐FU resistance and cell proliferation occurs partially through the regulation of two targets. As the first study to show that miR‐21 promotes resistance to 5‐FU in pancreatic cancer cells by targeting *PDCD4* and *PTEN*, our research demonstrates that a reduction in miR‐21 is critical to increase sensitivity to 5‐FU.

## Discussion

Most studies of pancreatic cancer focus on resectable cancers; however, few patients benefit from surgical resection because metastases are often present at the time of first diagnosis. Therefore, chemotherapy has become one of the most important treatments for pancreatic cancer 2. However, drug resistance is a major clinical problem for chemotherapy. As a common drug used in pancreatic cancer treatment, the mechanism of drug resistance to 5‐FU has not been well studied.

The study of miRNAs is growing rapidly, and recent studies have revealed an important role for miRNA in drug resistance. MicroRNA‐21 is commonly upregulated in many human cancers, including PDAC 16, 18. Recent studies also indicate that miR‐21 is related to drug resistance in various cancers, including breast cancer 18, glioblastoma cancer 19, and pancreatic cancer 20. However, most of the studies concerning the role of miR‐21 in drug‐resistant pancreatic cancer mainly concern gemcitabine resistance 16, 18, 21. There is small study on the role and mechanism of miR‐21 in 5‐FU resistance. To our knowledge, there are only two papers that report a relationship between miR‐21 and 5‐FU resistance in pancreatic cancer 14, 15. However, both of the studies are clinical investigations, and little is known about the mechanisms.

Our study explored the possible mechanisms of miR‐21‐induced 5‐FU resistance in pancreatic cancer cells. We inhibited the expression of miR‐21 in 5‐FU resistance cell line PATU8988/5‐FU and overexpressed miR‐21 in its parental cell line PATU8988 to test the function of miR‐21 in 5‐FU resistance, Our findings confirmed that resistance to 5‐FU in pancreatic cancer cells was associated with the overexpression of miR‐21. Introduction of miR‐21 into PATU8988 and PANC‐1 cells significantly enhanced their resistance to 5‐FU and also promoted the proliferation, migration and invasion of pancreatic cancer cells. Moreover, we explored the molecular pathways involved in 5‐FU resistance induced by miR‐21 and found that miR‐21‐induced 5‐FU resistance in pancreatic cancer mainly depends on the down‐regulation of its two tumor suppressor targets, *PTEN* and *PDCD4*. We also demonstrated the inhibitory role of *PTEN* and *PDCD4* in 5‐FU resistance in pancreatic cancer cells. *PDCD4* is a tumor suppressor gene involved in apoptosis, cell transformation, invasion and tumor progression. *PDCD4* exerts its activity by interacting with the eukaryotic translation initiation factors 4A (eIF4A) 22, 23, 24 and 4G (eIF4G) 25 to suppress mRNA translation and further inhibit the growth and proliferation of tumors 26. *PTEN*, a well‐known tumor suppressor gene, results in attenuation of PI3K/Akt/mTOR signaling pathway and tumor growth 27, 28. mTOR activates protein synthesis by phosphorylating key regulators of messenger RNA translation (including eIF4E) and ribosome synthesis. Messenger RNA translation in part is controlled by a eukaryotic initiation complex eIF4F (composed of eIF4E, eIF4G and eIF4A). So *PTEN* and *PDCD4* may have cross‐talk at protein synthesis.

Our study found that miR‐21 expression in PATU8988 and PANC‐1 cells results in a 2‐fold increase in the IC50 values of 5‐FU compared to the 28‐fold increase in the resistance index of PAT8988/5‐FU vs. parental PATU8988 cells. These results suggest that additional molecular factors may be involved in determining drug resistance in these cancer cells. One notable difference is that PATU8988/5‐FU cells had been treated with 5‐FU for 6 months.

The conclusion is that miR‐21 regulates 5‐FU drug resistance in pancreatic cancer by reducing the expression of its targets, *PTEN* and *PDCD4*. And *PTEN* and *PDCD4*, as tumor suppressors, not only can inhibit tumor growth and invasion, but also can downregulate the 5‐FU resistance induced by miR‐21 in pancreatic cancer cells. The study suggests that miR‐21 is a useful marker for therapeutic protocols in pancreatic cancer.

## Conflict of Interest

None declared.
